# Analysis of *PTEN* Methylation Patterns in Soft Tissue Sarcomas by MassARRAY Spectrometry

**DOI:** 10.1371/journal.pone.0062971

**Published:** 2013-05-17

**Authors:** Liang Yin, Wei-Juan Cai, Chun-Xia Liu, Yun-Zhao Chen, Jian-Ming Hu, Jin-Fang Jiang, Hong-An Li, Xiao-Bin Cui, Xiang-Yun Chang, Wen Jie Zhang, Kan Sun, Feng Li

**Affiliations:** 1 Department of Pathology and Laboratory of Xinjiang Endemic and Ethnic Diseases, Shihezi University School of Medicine, Shihezi, Xinjiang, P.R. China; 2 Department of Endocrinology and Metabolism, The First Affiliated Hospital, Shihezi University School of Medicine, Shihezi, Xinjiang, P.R. China; 3 Department of Clinical Laboratory, The First Affiliated Hospital, Shihezi University School of Medicine, Shihezi, Xinjiang, P.R. China; Vanderbilt University Medical Center, United States of America

## Abstract

Soft tissue sarcomas (STSs) are a rare and fascinating group of diseases that can be subdivided into specific reciprocal translocations in STSs (SRTSs) and nonspecific reciprocal translocations in STSs (NRTSs). PTEN mutations are rare in STSs, suggesting that PTEN expression may be lost by alternative mechanisms such as methylation. In order to reveal whether aberrant PTEN methylation occurs in STSs, MassARRAY Spectrometry was carried to detect methylation patterns of PTEN in STSs. We evaluated methylation levels in 41 CpG sites from −2,515 to −2,186 bp (amplicon A) and −1,786 to −1,416 bp (amplicon B) relative to the translation initiation site in 110 different cases (46 cases of SRTSs, 40 cases of NRTSs, and 24 cases of normal controls). In addition, immunohistochemistry (IHC) was used to detect the loss of PTEN to determine whether PTEN alterations were responsible for decreased PTEN expression. Our data showed that expression of PTEN was diminished in 49 (57%) STSs, whereas the remaining cases (43%) were classified as high expression. Our previous results found that only 2 of 86 cases (2.3%) had a PTEN mutation suggesting that PTEN may be mainly downregulated in STSs by methylation, but not by mutation of PTEN itself. We observed that amplicon A was hypermethylated in STSs with low PTEN expression, whereas normal controls had low methylation levels (*P*<0.0001), which was not present in amplicon B (*P*>0.05), nor were there significant differences in the methylation levels in PTEN between SRTS and NRTS cases. The majority of individual CpG units within two amplicons was demonstrated to be hypermethylated. These findings indicate that PTEN hypermethylation is a common event in STSs suggesting that the inactivation of PTEN may be due to hypermethylation in the promoter of PTEN. The aberrant methylation of the CpG sites within PTEN promoter may serve as a potential candidate biomarker for STSs.

## Introduction

Soft tissue sarcomas (STSs) comprise a heterogeneous group of mesenchymal tumors with wide spectrum of histologic features. More than 50 different subtypes have been proposed for STSs, in which malignant grades of tumors vary even among tumors of the same histologic categories [1]. Traditionally, sarcoma subtypes are further classified into two groups. One group is characterized by specific and balanced translocations, which include the integration of individual translocation genes (for example, alveolar rhabdomyosarcoma has PAX3-FKHR) and the other group typically shows more extensive chromosomal rearrangements leading to recurrent, but non-specific, chromosomal gains or losses [2]. Understanding the molecular pathogenesis of STSs may provide clues in developing therapeutic regimens for STSs. However, little information is available for STSs at molecular level in comparison with common cancers.

Phosphatase and tension homolog deleted on chromosome10 (PTEN) is a tumor suppressor and involved in basic cellular functions such as adhesion, migration, proliferation and cell survival [3]. PTEN is mutated in various tumors including STSs [4]–[6] but at a lower frequency in STSs than in other tumors. We have previously shown that PTEN mutation is not a frequent event in STSs [7], suggesting that other mechanisms may be involved in activating oncogenic pathways, such as epigenetic silencing due to promoter methylation which may play an important role in the descending process of PTEN expression. Thus, we favor the hypothesis that methylation in the PTEN promoter may underlie decreased PTEN expression in tumors without PTEN mutations. The promoter methylation of PTEN gene has been reported in tumors including STSs [8], [9], Suggesting possible involvement of DNA methylation in the pathogenesis of STSs.

Therefore, the aim of the present study is to quantitatively evaluate methylation status of CpGs within the PTEN promoter using MassARRAY Spectrometry and to determine whether aberrant PTEN promoter methylation occurs in STSs, whether methylation patterns affect expression levels of PTEN in sarcoma subtypes (SRTSs and NRTSs), and furthermore, whether any of these alterations has potential values serving as biomarkers of STSs.

## Materials and Methods

### Ethics Statement

Written informed consent was obtained from all participating patients before enrollment in the study. This study was approved by the institutional ethics committee at the First Affiliated Hospital of Shihezi University School of Medicine and conducted in accordance with the ethical guidelines of the Declaration of Helsinki.

### Tumor samples

We analyzed a total of 86 eligible formalin-fixed paraffin-embedded (FFPE) tissue samples from a collection of STS patients registered in the Departments of Pathology, Shihezi University School of Medicine and the People's Hospital of Xinjiang, China, during 1968 to 2010. The details of clinical features of patients with STSs are listed in Table 1. The diagnoses of all original slides including hematoxylin-eosin (HE) and immunohistochemistry staining from each case were confirmed by experienced pathologists review. Each paraffin block was reviewed to assure that at least 70% of the tumor cells were present before sectioning and DNA extraction. Patients with STS subtypes were composed of 46 cases of SRTSs (14 cases of Synovial sarcoma [SS], 6 cases of alveolar Rhabdomyosarcoma, 10 cases of Ewing's sarcoma, 10 cases of dermatofibrosarcoma protuberans [DFSP], and 6 cases of aveolar soft part sarcoma [ASPS]), and 40 cases of NRTSs (8 cases of leiomyosarcoma [LMS], 14 cases of malignant fibrous histocytoma [MFH], 6 cases of the embryonal Rhabdomyosarcoma, and 12 cases of myxofibrosarcoma [FS]) (See Table 1). Twenty-four (24) peripheral blood samples were collected in EDTA and used as normal controls.

**Table 1:** Relationship between the Expression of PTEN and Cliniopathological Features of Patients with Soft Tissue Sarcoma (n = 86).

Variable	Patients n (%)	PTEN Positive	PTEN Negative	*P* Value
Sex				0.2722
Male	52 (60.47)	25	27	
Female	34 (39.53)	12	22	
Age at diagnosis (yrs)				0.0574
0–20	20	2	18	
21–40	25	12	13	
41–60	22	9	19	
≥61	19	7	12	
Loation				0.9322
Extremity	25	12	13	
Head and neck	7	3	4	
Trunk wall	51	21	30	
Retroperitoneal	3	1	2	
Size (cm)				0.6803
<5	25	10	15	
5–10	27	13	14	
>10	23	8	17	
Missing	11	5	6	
Grade				0.8259
1	10(11.6)	4	6	
2	41(47.7)	15	26	
3	35(40.7)	17	18	
Histology				
SRTSs	46(53.)	19	27	0.6458
Synovial sarcoma	14(16.3)	5	9	
Alveolar rhabdomyosarcoma	6(7)	4	2	
Ewing's sarcoma	10(11.6)	3	7	
Dermatofbrosarcoma protuberans	10(11.6)	4	6	
Aveolar soft part sarcoma	6(7)	3	3	
NRTSs		15	25	0.9591
Leiomyosarcoma	8(9.2)	3	5	
Malignant fbrous histocytoma	14(16.3)	6	8	
Embryonal rhabdomyosarcoma	6(7)	2	4	
Myxofbrosarcoma	12(14)	4	8	

*P*-values were calculated by chi-square test. SRTSs, Specifc reciprocal translocations in STSs NRTSs, Nonspecifc reciprocal translocations in STSs.

### Isolation of genomic DNA

Total genomic DNA was extracted from the 86 FFPE samples and peripheral blood cells used as controls, using the standard phenol/chloroform extraction method [10]. Genomic DNA was stored at-20°C until used as template for each PCR reaction.

### PCR-SSCP analysis

The 4 exons of the PTEN gene were screened for mutations by PCR-SSCP and the procedures as described previously [7].

### Immunohistochemistry (IHC)

For all STS samples, 2-µm thick sections were cut from the paraffin blocks and deparaffinized by routine techniques followed by microwave treatment for 10 min in EDTA buffer (pH 9.0). After cooling, slides were incubated with monoclonal antibody against human PTEN (1∶100, #9559; Cell Signaling Technology, Beverly, MA, USA), using an automated staining device (DAKO, Carpinteria, CA, USA) with a dilution of 1∶50. Primary antibody was detected by biotinylated secondary antibody followed by avidin–biotin peroxidase complex and 3,3′-diaminobenzidine chromagene. Finally, sections were counterstained by hematoxylin. To obtain negative controls, the primary antibody was omitted.

The immunostaining patterns and intensities were evaluated independently by two pathologists. PTEN expression in tumor cells was compared with that in vascular endothelial cells that are used as an internal positive controls. Scoring was performed according to the percentage of positive cells: <5% was classified as negative (−), 6–100% was classified as positive. 6–30% of positive cells were scored with +, 31–60% with ++, >60% with +++. A blinded repeat test produced similar results.

### MassARRAY analysis

**Figure 1:**
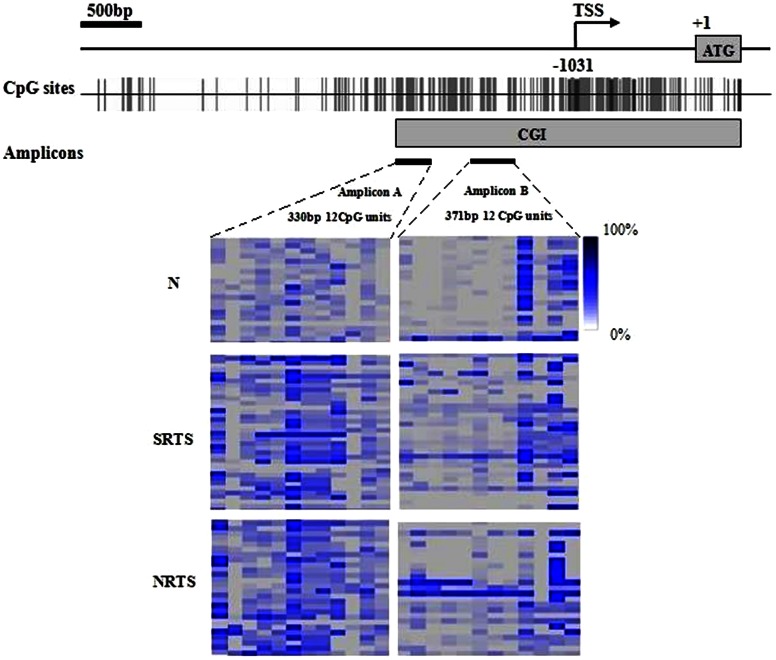
Genomic map of PTEN CGI and the DNA methylation status at 2 different amplicons of the PTEN. MassARRAY analysis is used to quantify DNA methylation of PTEN in STS tissues and normal controls. Arrow indicates the transcriptional start site (TSS) at −1031 bp. ATG, translation start codon, +1 indicates the translation initiation site. Vertical bars, CpG sites. Gray filled boxs, CGI; Black filled bars, MassARRAY regions studied [Amplicon (A), (B)], amplicon characteristics are shown beneath the black bars; bp indicates base pair. Each row represents a sample. Top, 20 normal controls (N); middle, 34 cases of SRTSs, and bottom, 26 cases of NRTSs. Each column represents a CpG unit, which is a single CpG site or a combination of CpG sites. Color coding reflects the degree of methylation with dark blue being 100% and white being 0%; gray, no data.

The sequence of CpG island (CGI) was identified by the use of the UCSC genome browser (http://genome.ucsc.edu/), (chr10:89621773-89624128 %GC = 58.1 and Obs/Exp CpG = 0.86). The targets of the promoter regions were two sites (Fig. 1): amplicon A, amplicon B, as previously reported [11], [12], [13]. We designed two primer sets for methylation analysis of the PTEN promoter region by EpiDesigner software (http://epidesigner.com; Table 2). For each reverse primer, an additional T7 promoter tag was added for in vivo transcription, and a 10-mer tag was added to the forward primer to adjust for the melting temperature differences. The primers used in the present study detected specifically the promoter sequence of the PTEN gene rather than that of the PTEN pseudogene [14].

**Table 2:** Sequences of MassARRAY primers and positions relative to the translational start codon for the assays used to analyze DNA methylation of PTEN (2 amplicons).

Amplicon	Primer	Sequence (5′→3′)	Position
Amplicon A	PTEN-1F	aggaagagagTGAGTTTTAGGTTTTAGTTTTTTGGTTTGT 3	−2,515 to −2,186
	PTEN-1R	cagtaatacgactcactatagggagaaggctTTAAAAAACTTTCCAAATTCCCACTCC 3′	
Amplicon B	PTEN-2F	aggaagagagTTAGATAGGTGTTTTTTGGGTTTTTGA	−1,786 to −1,416
	PTEN-2R	cagtaatacgactcactatagggagaaggctTCCCTACAAAAAAAATACCCTCCCC	

Quantitative DNA methylation analysis of PTEN using the MassARRAY platform (SEQUENOM) was carried out as described previously [15]. Briefly, 1 µg of genomic DNA was treated with sodium bisulfite, PCR amplified, two bisulfite reactions were designed, which covered 21,and 20 CpGs, respectively, and extend from −2,515 to −2,186 bp (amplicon A) and from-1,786 to −1,416 bp (amplicon B) relative to the translation initiation site (Figure 1). In vitro transcribed, and then cleaved by RNase A.The samples were then quantitatively tested for their DNA methylation status using matrix-assisted laser desorption ionization-time of flight mass spectrometry. Methylation data of individual units (one to three CpG sites per unit) were generated by the EpiTyper v1.0.5 software (SEQUENOM). The non-applicable reading and its corresponding site were eliminated in calculation.

### Statistical Methods

Statistical analysis was performed using Fisher's Exact test and Wilcoxon test (two-sided) in GraphPad Prism version 5.0 (GraphPad Software, Inc., La Jolla, CA). Correlation between experimental data and clinical data were also performed. Statistical analysis of the different methylation level between groups was performed using a two-way ANOVA. All values were represented as mean±SD. Significance was set as a *P* value of <0.05.

## Results

### PTEN expression in STSs

**Figure 2:**
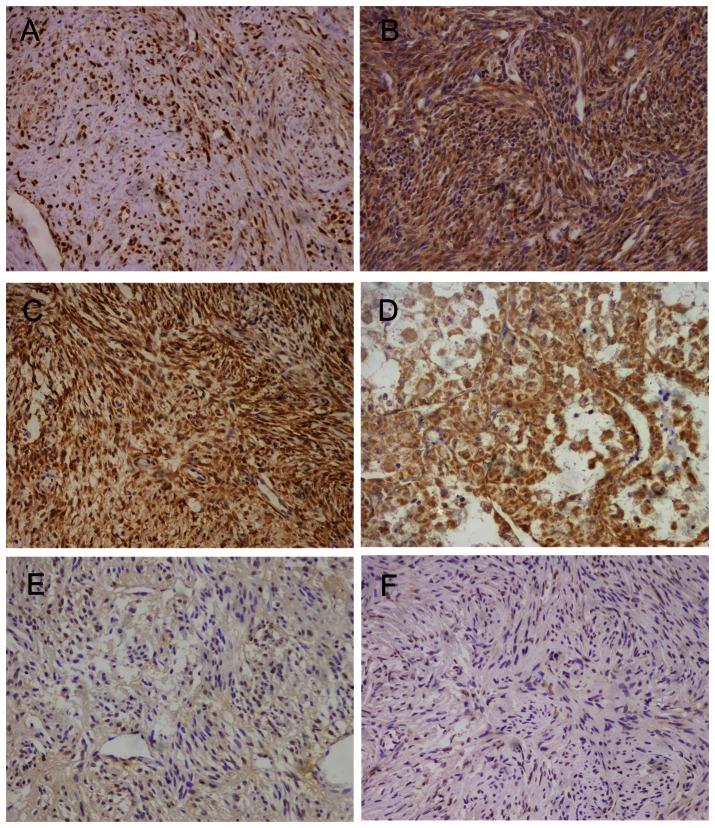
Immunohistochemical analysis of PTEN in STS. Positive immunohistochemical staining in (A) leyomiosarcoma, (B) Synovial sarcoma, (C) Dermatofbrosarcoma protuberans,and (D) Alveolar rhabdomyosarcoma. Negative immunohistochemical staining in (E) high-grade leyomiosarcoma and (F) Dermatofbrosarcoma protuberans. All Original magnification×20.

In the present study, the immunohistochemistry was used to detect the loss of the PTEN to determine whether PTEN gene alterations are involved in decreased PTEN expression.Our datas show that 49 (57%) of the 86 STSs cases revealed decreased expression of PTEN protein. Staining was observed both in cytoplasm and in nucleus of tumor cells. Fig. 2 shows some examples of typical positive stainings. There were no significant differences in expression levels between specific reciprocal translocations in SRTSs and NRTSs,41.30% and 37.5%,respectively. The realationship between PTEN expression and clinical Features were evaluated. There were no significant association between PTEN expression and age, gender, tumor size, location of the primary tumor ,tumor grade and different histotypes,as shown in Table 1.

### Mutation analysis of thePTEN genes by PCR-SSCP

All 86 STSs were screened by PCR-SSCP for PTEN mutations in exons 5 to 8. Only 2 out of 86 STSs (2.3%) had an aberrant SSCP shift in exon 8 of PTEN. Two cases with PTEN gene mutations were shown to be histological ASPS and MFH, respectively (Table 3).

**Table 3:** Summary of PTEN mutation and correlation between histological subtypes and protein expression and promoter methylation of the amplicon A in Soft Tissue Sarcoma cases.

Histological subtypes	Protein expression		PTEN mutation	*Amplicon A Methylation*	
	*Negative*	*Positive*		H	U
SRTSs					
Synovial sarcoma	6	3	0	8	1
Alveolar rhabdomyosarcoma	3	3	0	4	2
Ewing's sarcoma	7	3	0	5	5
Dermatofbrosarcoma protuberans	6	4	0	7	3
Aveolar soft part sarcoma	2	1	1	2	1
Total	24	14		26	12
*P*-value	0.76			0.50	
NRTSs					
Leiomyosarcoma	3	3	0	4	2
Malignant fbrous histocytoma	4	6	1	6	4
Embryonal rhabdomyosarcoma	2	2	0	3	1
Myxofbrosarcoma	2	0	0	2	0
Total	11	11		15	7
*P*-value	0.49			0.72	

*P*-values were calculated by chi-square test. U, unmethylated; H, hypermethylated.

### Frequent Epigenetic silencing of PTEN in STSs

We carried to detect methylation Patterns of PTEN CpG island and performed quantitative high throughput analysis of DNA methylation by the MassARRAY system within the PTEN −2,515 bp to −2,186 bp (amplicon A) and −1,786 bp to −1,416 bp (amplicon B) relative to the translation initiation site, which contains 41 CpG sites (Figure 1, Table 2). The methylation status of PTEN promoters were studied in all the samples collected from STSs (n = 86) and normal controls (n = 24). A 330 bp region of the PTEN promoter containing 21 CpG sites which could be divided into 14 CpG units and a 371 bp region of the PTEN promoter containing 20 CpG sites which could be divided into 16 CpG units were examined by MassARRAY system. Among these units, 6 CpG units (10 CpG sites) did not yield successful measurements. Sixty (60) samples had good results for >90% of the samples. The final data set consisted of 24 CpG units from 60 samples. The average DNA methylation frequency ranged from 0.0%–51.0% in STSs (PTEN methylation ranged from 0.0%–43.6% in STRS and from 0.7%–51.0% in NRTS.) and from 0.4%–7.7% in normal controls.

**Figure 3:**
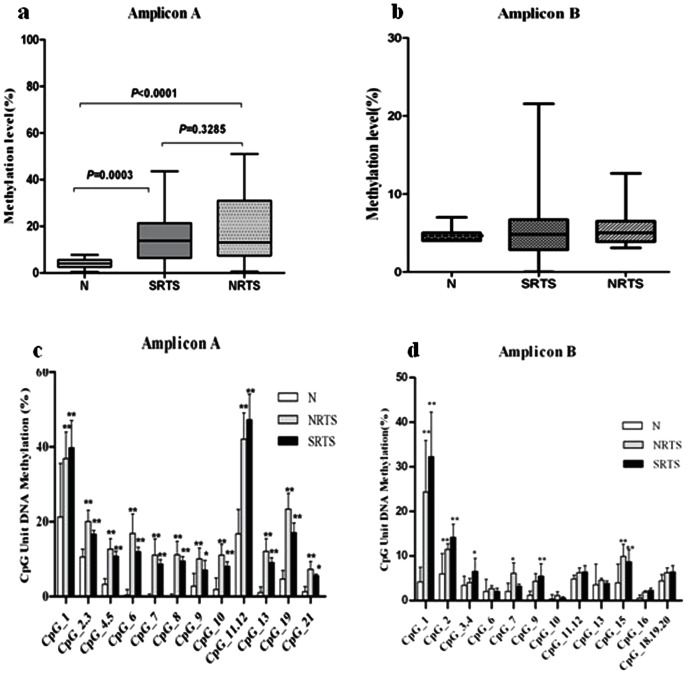
The methylation levels of STSs are displayed within amplicon A and amplicon B. (**a,b**)The overall methylation levels are displayed within amplicon A and amplicon B as box plots in SRTSs and NRTSs compared with normal controls.(c,d)The average methylation of the CpG units of amplicon A and amplicon B is presented for SRTSs, NRTS, and normal controls. SRTSs or NRTSs carrier compared with normal controls: * *P*<0.05 and ** *P*<0.001. Error bars represent standard error.

“Hypermethylation” is defined as a test sample's average methylation across all CpGs measured is beyond 3 standard deviations (SD) from the mean derived from 20 normal individuals (“mean of controls + 3×SD” [16]). Aberrant methylation was significantly higher within amplicon A of the PTEN promoter in SRTSs and NRTSs than in normal controls, an average of 26%, 23% and 4% (Figure 3 a), respectively. In contrast, there were no significant methylation changes observed within amplicon B in SRTSs and NRTSs than in normal controls (*P*>0.05, Figure 3b). No association between PTEN methylation and age of the patients or stage of disease was found. These datas demonstrate the occurrence of aberrant promoter methylation of PTEN in STS samples and indicate that the PTEN promoter is the most abundantly methylated in STSs.

We used an unsupervised two-way hierarchical clustering of the CpG unit methylation and the STSs and normal controls (Figure 1). The patterns we observed in the cluster analyses show that methylation patterns of normal controls are notably different from those observed in tumor tissues.

### Methylation levels at individual CpG Sites along PTEN promoter region

We next examined the methylation status of individual CpG sites within each promoter region (Figure 3c and 3d). For PTEN, there was some variability in the methylation level of individual CpG sites within the sequenced region. For amplicon A, all CpG units showed significant methylation differences in SRTSs and NRTSs compared to normal controls (Figure 3c). The methylation level of almost every CpG units was numerically higher in samples of NRTSs, except in CpG_1 and CpG_11.12 were significantly different between these two groups(*P*<0.001). Even though there were no differences in overall methylation within amplicon B in SRTSs and NRTSs than in normal controls, we found heterogeneity between individual CpG units. Six CpG units showed increased methylation in STSs relative to normal controls (CpG_1, CpG_2, CpG_3.4, CpG_7, CpG_9, CpG_15) (Figure 3d). Moreover, we also observed that CpG_1was significantly different between SRTSs and NRTSs (*P*<0.001).

### Correlation Between Promoter Methylation and the Expression of PTEN

To further evaluate whether PTEN promoter amplicon A methylation correlated with PTEN protein expression. Among 60 STS cases, 41 cases were found to be hypermethylated whereas 19 cases were identified as unmethylated. Of 41 hypermethylated cases, 28 (68%) cases were seen as immunoreaction negative whereas of 19 samples negative for PTEN promoter methylation, loss of PTEN expression was only observed in 7 (37%), suggesting an inverse association betweenPTEN promoter methylation and PTEN protein expression (Fisher's exact test, P = 0.027) (Table 4). Nevertheless, there was no significant correlation between histological subtypes and protein expression, promoter methylation of the PTEN gene (SRTSs;*P* = 0.76 and 0.50, NRTSs;*P* = 0.49 and 0.72, Fisher's exact test, respectively) (Table 3).

**Table 4:** Correlation Between Promoter Methylation and the Expression of PTEN **in** Soft Tissue Sarcomas (n = 60 cases).

*Amplicon A Methylation*	PTEN Expression		*P-value*
	*Negative*	*Positive*	
Hypermethylated	28	13	0.0273*
Unmethylated	7	12	

* Statistically significant.

## Discussion

Most STSs have no clearly defined etiology although multiple association or predisposing factors have been identified [17]. Morerover, this disease remains at high risk for recurrence and death. As for other cancers, genetic factors play a crucial role in the initiation and progression of sarcomas. Tumor suppressor genes play a critical role in cell growth inhibition and can suppress the growth of cancer cells. The PTEN tumor-suppressor gene is mutated in diverse human cancers [18]. PTEN mutation occurs at a lower frequency in STS than in other tumors and therefore, epigenetic modifications of DNA, such as DNA methylation, are hypothesized to play a key role in STSs progression.

Studies to date have suggested a low frequency of PTEN mutation in STSs. For example, Kawaguchi et al. has reported a frequency of PTEN methylation in only 12% of soft tissue sarcomas studied and concluded that promoter methylation of the PTEN gene is a relatively rare event in STS [8]. Another study by Li et al. [19] has suggested that the methylation does not affect PTEN expression in cell lines which express PAX3 or PAX3 and PAX3-FKHR in rhabdomyosarcoma tumourigenesis.

In the current study and for the first time, we have employed MALDI-TOF MS to evaluate methylation patterns at multiple CpG sites within the promoter region of PTEN in STSs. Our findings indicate that aberrant methylation of PTEN is significantly higher in STSs than in normal controls. When two amplicons specific for PTEN CpG islands are analyzed by MALDI-TOF MS, the methylated CpG islands appear to be a dominant mechanism in the sarcomas studied. We have previously reported the promoter methylation of PTEN in 24.5% of sarcomas, but PTEN gene mutation only in 2.3% of cases [7]. Here we show that aberrant methylation is significantly higher within amplicon A of the PTEN promoter in both SRTSs and NRTSs than that in normal controls, but there are no significant differences in methylation observed within amplicon B (*P*>0.05). Although our results did not show association between methylation status and the subtypes of soft tissue sarcomas, we found a significant inverse association between amplicon A methylation and PTEN protein expression suggesting that the methylated promoter may be a mechanism leading to inactivation of the PTEN gene.

We have further evaluated aberrant methylation status of CpG units that may be used as novel biomarkers for STSs. Previous studies suggest that quantitative cytosine methylation profiling can be used to identify molecular markers in tumors [20]–[23]. These studies have revealed specific hypermethylated CpG sites that are useful in diagnosis of cancers. In this study, we have shown that significant differences in the frequency of methylation at individual CpG units between STSs and normal controls. For amplicon A, all CpG units (CpG_6, CpG_11.12 and CpG_19) are significantly hypermethylated in STS cases as compared with controls (*P*<0.001). Meanwhile in amplicon B, 6 CpG units show significantly increased methylation in STS cases compared with normal controls. Moreover, we have also observed that CpG_1 is significantly different between SRTS cases and NRTS cases (*P*<0.001). Thus, these observations suggest a possibility that the methylation frequency at individual CpG units might serve as novel diagnostic biomarkers capable of distinguishing among tissues of normal individuals, SRTS cases and NRTS cases.

In this study, the methylation status of PTEN gene has not been able to show a correlation with clinical prognosis and/or AJCC stages in patients with STSs, which may be due to the limited sample size of this study. However, the current investigation has paved the way for studying PTEN gene methylation using MALDI-TOF MS technology and warrant further studies using larger sample sizes of STSs.

In conclusion, this is the first report analyzing PTEN promoter methylation using MALDI-TOF MS technology and the results indicate that promoter hypermethylation of the PTEN gene is a common event in STSs which may play a role in the oncogenesis of STSs. The aberrant methylation in CpG sites within the PTEN promoter may potentially serve as a candidate biomarker for STSs.
